# MRGBP as a potential biomarker for the malignancy of pancreatic ductal adenocarcinoma

**DOI:** 10.18632/oncotarget.19451

**Published:** 2017-07-22

**Authors:** Feng Ding, Shuang Zhang, Shaoyang Gao, Jian Shang, Yanxia Li, Ning Cui, Qiu Zhao

**Affiliations:** ^1^ Department of Gastroenterology/Hepatology, ZhongNan Hospital of Wuhan University, Wuhan 430071, China; ^2^ The Hubei Clinical Center & Key Laboratory of Intestinal & Colorectal Diseases, Wuhan 430071, China; ^3^ Laboratory of Clinical Immunology, Wuhan No.1 Hospital, Tongji Medical College, Huazhong University of Science and Technology, Wuhan 430022, China; ^4^ Department of Pathology, Hubei Cancer Hospital, Wuhan 430079, China; ^5^ Department of Gastroenterology, Renmin Hospital of Wuhan University, Wuhan 430060, China

**Keywords:** MRGBP, pancreatic ductal adenocarcinoma, biomarker, upregulation

## Abstract

MORF4-related gene-binding protein (MRGBP), which is also known as chromosome 20 open reading frame 20 (C20orf20), is commonly highly expressed in several types of malignant tumors and tumor progression. However, the expression pattern and underlying mechanism of MRGBP in pancreatic ductal adenocarcinoma (PDAC) remain unknown. In the study, we found that MRGBP was frequently upregulated in PDAC tissues and cell lines. In addition, the upregulation of MRGBP was positively associated with TNM stage, T classification, and poor prognosis. Knockdown of MRGBP in the PDAC cell lines ASPC-1 and Mia PaCa-2 by transiently transfected with small interfering RNA (siRNA) drastically attenuated the proliferation, migration, and invasion of those cells, whereas ectopic MRGBP overexpression in BxPC-3 cells produced exactly the opposite effect. Furthermore, we also found that overexpression of MRGBP remarkably led to cell morphological changes and induced an increased expression of mesenchymal marker Vimentin, whereas a decreased expression of epithelial marker E-cadherin. Taken together, this study indicates that MRGBP acts as a tumor oncogene in PDAC and is a promising target of carcinogenesis.

## INTRODUCTION

Pancreatic cancer (PC), which is the seventh most common cause of cancer-related deaths worldwide [1], is expected to become the second cause of cancer-related deaths by 2030 [2]. In contrast to the steady increase in survival for most cancers, advance has been slow for pancreatic cancer, for which the 5-year relative survival is currently 7% [3]. Pancreatic ductal adenocarcinoma (PDAC) constitutes 90% of pancreatic cancer. Surgical resection remains the only potentially curative treatment. However at the time of diagnose, most patients present with metastasis to the regional lymph nodes and distant organs [4]. The molecular mechanism of PDAC is extraordinarily perplexed and heterogeneous that is accompanied by various genetic abnormal expressions. Even though various gene signatures and signaling pathways have been discovered in recent years, the PDAC pathogenesis has not completely clarified until now. Facing to the fast-growing morbidity and the high mortality, identification of potential therapeutic targets and diagnostic biomarkers for PDAC is urgently needed.

MORF4-related gene-binding protein (MRGBP), which is also known as chromosome 20 open reading frame 20 (C20orf20), is identified as a protein capable of binding directly to MRG15 and MRGX proteins that are two indispensable components of tat-interacting protein 60 (TIP60) histone acetyltransferase (HAT) complex and histone deacetylases (HDACs) complexes [5]. The abnormally expressed TIP60/HAT complex and/or HDACs complexes have been shown to be associated with cell differentiation, proliferation, migration and invasion in diverse human cancers [6–8].

It has been demonstrated that MRGBP was upregulated in multiple types of cancer such as colorectal cancer, cervical cancer, prostate cancer, and cutaneous squamous cell carcinoma, and has been proved to increase replication, induce apoptosis, reduce growth and promote invasiveness [9–12]. Expression of MRGBP has been shown to play an important role in regulating stability and/or synthesis of MRG15 and MRGX [13]. These discoveries implied that MRGBP may have biological roles as diagnostic biomarker and anticancer target for aforementioned cancers. However, little is known about the expression pattern, clinicopathologic significance and functional role of MRGBP in PDAC.

In current study, we showed, for the first time, the experssion of MRGBP is upregulated in PDAC. Furthermore, we discovered that upregulated expression of MRGBP was associated with poor prognosis in patients with PDAC. What's more, we also found knockdown of MRGBP suppressed the proliferation, migration and invasion in PDAC cells. In addition, we also demonstrated that overexpression of MRGBP promotes poliferation and induces EMT in PDAC cells. Taken together, our findings suggest that MRGBP can serve as a novel biomarker for PDAC and the development of diagnostic and/or therapeutic approaches.

## RESULTS

### MRGBP is highly expressed in PDAC tissues and PDAC cell lines

**Figure 1:**
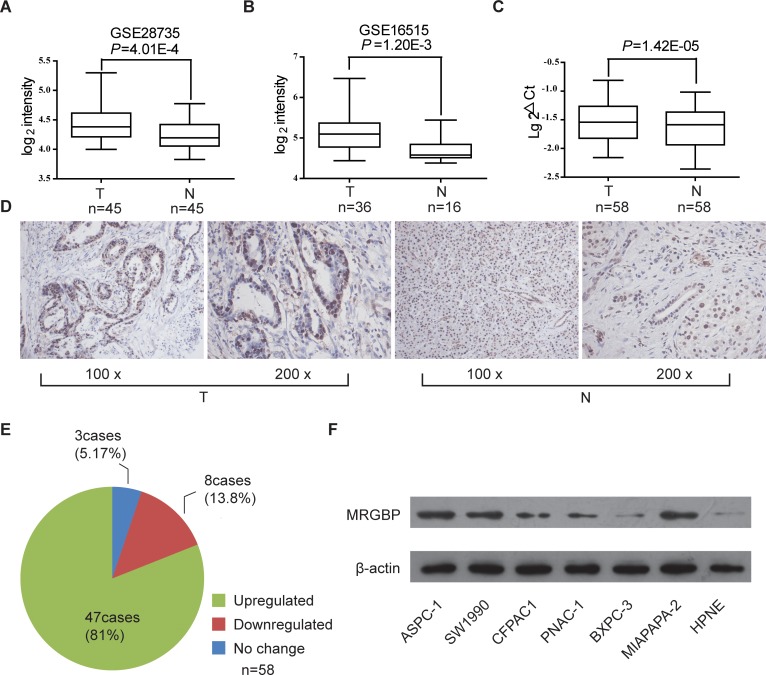
The expression of MRGBP is increased in pancreatic ductal adenocarcinoma (PDAC) **(A)** MRGBP expression level in PDAC tissues (T) and adjacent noncancerous tissues (N) in dataset GSE28735. The paired t-test was used. **(B)** MRGBP expression level in PDAC tissues and normal pancreas in dataset GSE16515. The paired t-test was used. **(C)** The mRNA expression level of MRGBP in 58 paired tissues of PDAC and adjacent tissue were analyzed by real-time PCR. The paired t-test was used. **(D)** Representative photographs of staining of MRGBP protein in a pair of PDAC and adjacent tissues. **(E)** The expression of MRGBP was upregulated in 81.0% of PDAC patients examined by immunohistochemical staining. **(F)** The protein levels of MRGBP were assessed in 6 PDAC cell lines as well as a normal cell line (HPNE) by Western blotting analysis.

In order to illustrate the biological expression pattern of MRGBP in PDAC, we observed the mRNA level of MRGBP in the large cohorts of PDAC patients available from two independent GEO datasets GSE28735 and GSE16515. The results showed that MRGBP expression was highly expressed in PDAC tissues comparing with paired adjacent noncancerous pancreatic tissues using GSE28735 (Figure 1A, n = 45, p = 4.01E-4). In addition, expression of MRGBP was also significantly up-regulated in the PDAC tissues than the normal pancreas using GSE16515 (Figure 1B, p = 4.29E-5). In present study, MRGBP expressions of 58 paired PDAC and non-cancerous tissues were investigated by quantitative real-time PCR (qRT-PCR). The results indicated that PDAC tissues exhibited significant upregulation of MRGBP compared with non-cancerous tissues (Figure 1C, p = 1.42E-05). To further address the role of MRGBP in PDAC, the protein expression of MRGBP was detected in 58 paired PDAC and non-cancerous tissues using immunohistochemical staining. As shown in Figure 1D, MRGBP was expressed in nucleus. The expression of MRGBP protein was also pronounced elevated in the majority of PDAC patients (Figure 1E, 81.0%, 47/58).

In addition, MRGBP expression was examined by western blot in different PDAC cell lines, including HPNE, PANC-1, SW1990, Mia PaCa-2, AsPC-1, BxPC-3, CFPAC1. In accord with the findings from tissues, significantly upregulated expression of MRGBP was detected in most PDAC cells, compared with HPNE cells (Figure 1F). These findings evidently support the notion that MRGBP expression was remarkably upregulated in PDAC on both mRNA and protein level, implying the importance of it in PDAC pathogenesis.

### Correlation between MRGBP expression and several clinicopathologic characteristics in patients with PDAC

To determine the clinicopathological significance of MRGBP expression in PDAC, we performed immunohistochemistry (IHC) analysis to evaluate its expression using 58 PDAC cases that underwent curative surgical resection. As shown in Table 1, MRGBP expression in PDAC tissues was strongly associated with TNM stage (p = 0.042) and T classification (p = 0.028), but not with other parameters including age, gender, tumor location, tumor size lymph node metastasis, distant metastasis, vascular invasion and histological differentiation. These data supported that upregulated MRGBP could represent a potential new prognostic factor for PDAC and might contribute to tumor progression in PDAC.

**Table 1:** Correlations between MRGBP expression and the clinicopathologic features in patients with PDAC

Characteristics	Total58	Expression of MRGBP		P value
		High (n=29)	Low(n=29)	
Age, years				
≤65	27	12	15	0.430
>65	31	17	14	
Gender				
Male	33	18	15	0.633
Female	25	11	14	
Tumor location				
Head	42	23	19	0.240
Body/tail	16	6	10	
TNM (AJCC)				
I	11	2	9	0.042
II	38	20	18	
III	6	4	2	
IV	3	3	0	
Tumor size, d/cm				
≤3.5	26	11	15	0.291
>3.5	32	18	14	
T classification				
T1-2	13	3	10	0.028
T3-4	45	26	19	
Lymph node metastasis				
-	37	16	21	0.172
+	21	13	8	
Distant metastasis				
-	55	26	29	0.236
+	3	3	0	
Vascular invasion				
-	51	24	27	0.420
+	7	5	2	
Histological grade				
Well	6	2	4	0.666
Moderate/poor	52	27	25	
CA19-9(ng/mL)				
≤150	29	13	16	0.431
>150	29	16	13	

### High expression level of MRGBP predicts poor prognosis of PDAC patients

**Figure 2:**
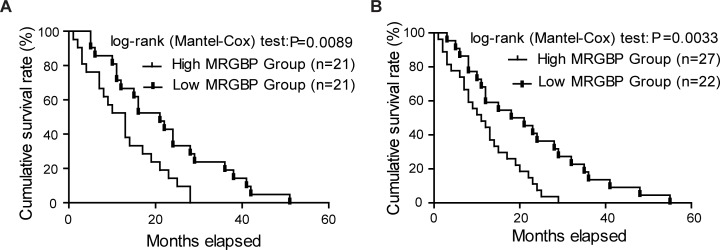
Overexpression of MRGBP is correlated with poor prognosis of pancreatic ductal adenocarcinoma (PDAC) **(A)** The correlation between MRGBP expression and patient survival was conducted in dataset GSE28735. **(B)** Overall survival analysis of PDAC patients with different MRGBP expression in 49 enrolled fatal PDAC cases.

To evaluate the prognostic value of MRGBP in patients with PDAC, the relationship between the expression level of MRGBP and the overall survival (OS) was evaluated by the Kaplan-Meier analysis and log-rank test. We first determined the prognostic value of MRGBP at mRNA level using the independent GEO datasets GSE28735. Three specimens without survival time were excluded from study. The results showed that the overall survival of patients with high levels of MRGBP was significantly poorer than patients with low levels of MRGBP protein levels (median survival 13 months in the high-expression group vs. 21 months in the low-expression group, p = 0.0089) (Figure 2A).

The 58 PDAC patients enrolled were divided into two groups equally according to the expression level of MRGBP. Furthermore, among the 58 cases there were 49 deaths, of which 27 were from high MRGBP expression group. To reinforce the persuasiveness of the above results, we evaluated the prognostic significance of MRGBP in the 49 fatal cases enrolled. As expected, we also found that higher MRGBP expression was significantly associated with decreased overall survival (Figure 2B, p = 0.0033). Overall, these data above strongly suggested that high expression of MRGBP hints poor prognosis in PDAC.

### Downregulation of MRGBP inhibits proliferation and causes apoptosis in PDAC cells *in vitro* and *in vivo*

**Figure 3:**
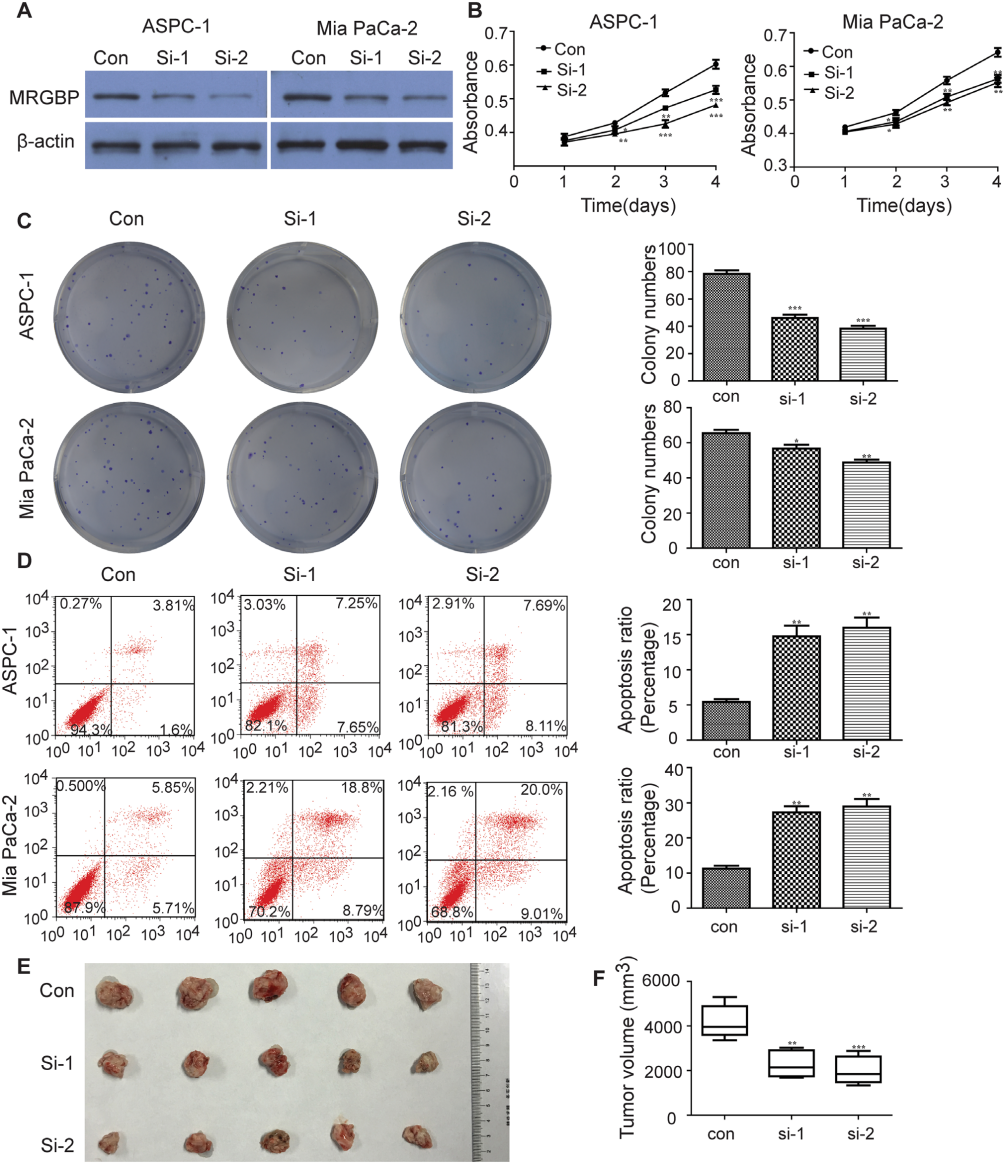
Knockdown of MRGBP inhibits proliferation and causes apoptosis in PDAC cells *in vitro* and *in vivo* **(A)** Knockdown of MRGBP in ASPC-1 and Mia PaCa-2 cell lines by siRNAs was confirmed by Western blotting. **(B)** Effect of MRGBP knockdown on the proliferation on ASPC-1 and Mia PaCa-2 cells was determined by CCK-8 assay. **(C)** Effect of MRGBP knockdown on colony numbers was determined by colony formation assay in ASPC-1 and Mia PaCa-2 cells. **(D)** Effects of MRGBP knockdown on ASPC-1 and Mia PaCa-2 cells apoptosis were determined by flow cytometry. **(E)** Tumors were dissected, measured and exhibited in each group. **(F)** Representative data showed that downregulation of MRGBP significantly inhibited tumor growth. *, **, *** represents P < 0.05, P < 0.01, P < 0.001 respectively.

Considering the close correlation between MRGBP expression and the poor prognosis, the biological process potentially modulated by MRGBP should be investigated. To determine the biological effect of MRGBP in proliferation of PDAC, MRGBP expression was inhibited by siRNAs in ASPC-1 and Mia PaCa-2, which exhibit a higher expression of MRGBP. Stable expression of two siRNA targeting MRGBP resulted in remarkable decrease in MRGBP expression of ASPC-1 and Mia PaCa-2 cells (Figure 3A). As expected, CCK-8 assays showed that MRGBP knockdown inhibited cell proliferation in ASPC-1 and Mia PaCa-2 cells (Figure 3B). Similar results were observed in plate colony formation assay. MRGBP knockdown significantly inhibited colony formation of ASPC-1 and Mia PaCa-2 cells compared with the si-Ctrl group (Figure 3C). Meanwhile, cell apoptosis assay was employed to investigate the biological function of MRGBP in apoptosis of PDAC by flow cytometric analysis. As shown in Figure 3E, silencing of MRGBP significantly increased the numbers of apoptotic cells in ASPC-1 and Mia PaCa-2 cells (Figure 3D). Additionally, to explore MRGBP-mediated effects *in vivo*, xenograft tumor assay was performed using transfected Mia PaCa-2 cells. Indeed, tumor volumes obtained from si-1 and si-2 groups were significantly smaller than that from control group (Figure 3E, 3F). This experiment suggests that si-MRGBP significantly inhibits xenogratf tumor growth in nude mice. Taken together, these results clearly indicated that downregulation of MRGBP inhibits the proliferation and tumorigenicity of PDAC cell.

### Upregulation of MRGBP promotes PDAC cell proliferation *in vitro*

**Figure 4:**
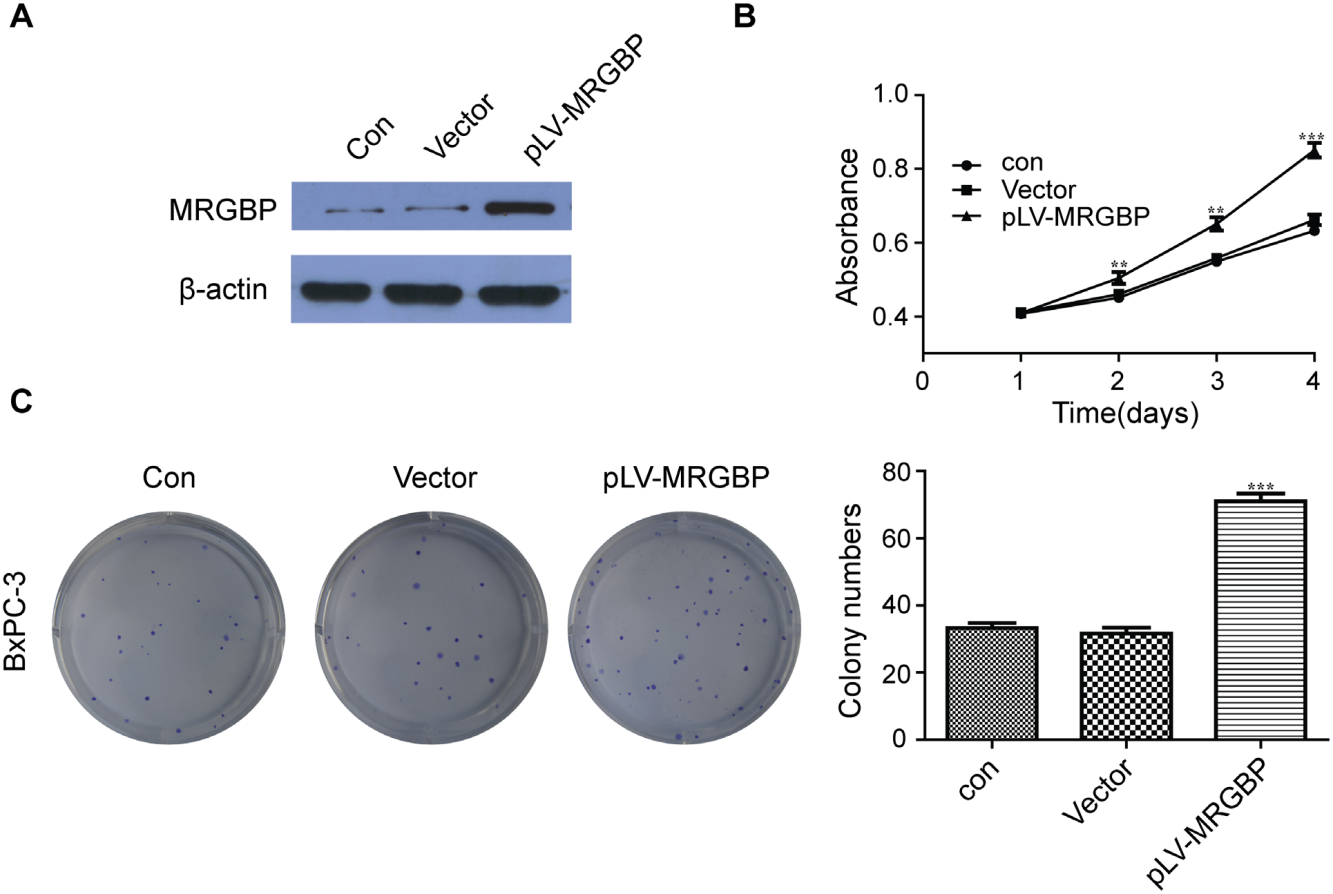
Overexpression of MRGBP promotes PDAC cell proliferation **(A)** Overexpression of MRGBP in BxPC-3 cell line was confirmed by Western blotting. **(B)** Effect of MRGBP overexpression on the proliferation on BxPC-3 cells was determined by CCK-8 assay. **(C)** Effect of MRGBP overexpression on colony numbers was determined by colony formation assay in BxPC-3 cells.

To further verify the positive role of MRGBP in PDAC cell proliferation, MRGBP was also stably upregulated by lentivirus-mediated packed pLV-MRGBP vector in BxPC-3 cell line. The overexpressed MRGBP in cells were affirmed by Western blot (Figure 4A). Naturally, the same methods were used to evaluate the effect of MRGBP overexpression on PDAC cell proliferation. As expected, MRGBP-overexpressing BxPC-3 cells displayed a significantly higher cell growth rate than the empty-vector cells (Figure 4B). In addition, cell proliferation was also measured by using the plate colony formation assay. Similarly, compared to the empty-vector cells, BxPC-3 cells with overexpressed MRGBP showed markedly increased colony formation (Figure 4C, 4D). Collectively, these results demonstrated that overexpression of MRGBP promotes PDAC cell proliferation.

### MRGBP modulates cell migration and invasion of PDAC cells *in vitro*

**Figure 5:**
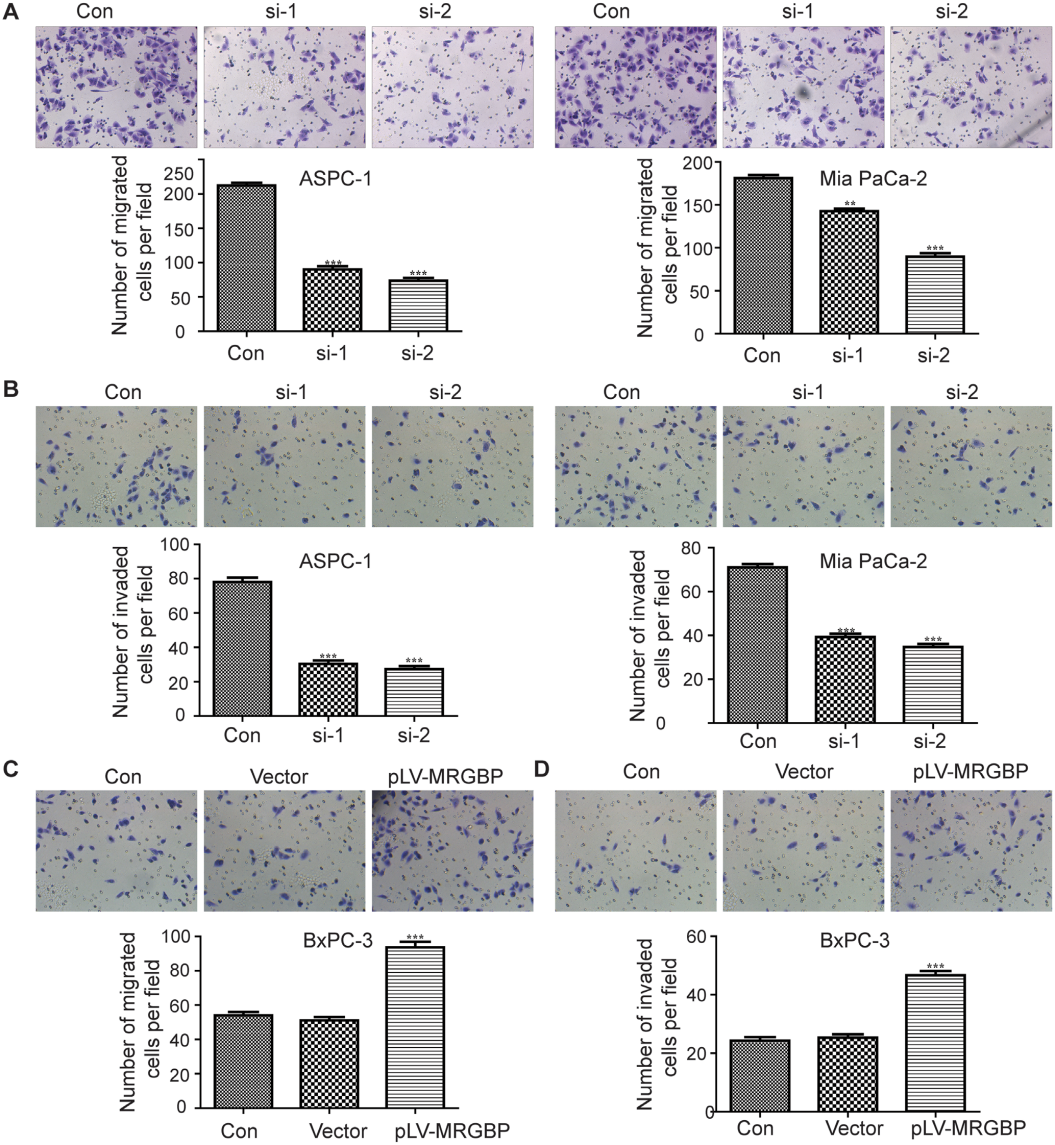
MRGBP modulates cell migration and invasion of PDAC cells *in vitro* **(A)** Migration capability was evaluated by transwell assay in ASPC-1 (left) and Mia PaCa-2 (right) cells after the cells were transfected with MRGBP siRNA. **(B)** Invasion capability was assessed by transwell assay with matrigel in ASPC-1 (left) and Mia PaCa-2 cells (right) after the cells were transfected with MRGBP siRNA. **(C)** Migration capability was evaluated by transwell assay in BxPC-3^vector^ and BxPC-3^MRGBP^ cells. **(D)** Invasion capability was assessed by transwell assay with matrigel in BxPC-3^vector^ and BxPC-3^MRGBP^ cells.

The role of MRGBP in PDAC cell proliferation is in agreement with the previous reports in colorectal cancer and cutaneous squamous cell carcinoma [9, 12]. Furthermore, our research suggested that increased MRGBP expression in PDAC tissues was strongly associated with high TNM stage and poor prognosis, but not with lymph node metastasis, distant metastasis, vascular invasion. Therefore, transwell assays were employed to evaluate whether MRGBP has impact on cell migration and invasion. The results showed that knockdown of MRGBP significantly suppressed the migration and invasion of ASPC-1 and Mia PaCa-2 cells (Figure 5A, 5B). Whereas, upregulation of MRGBP had the opposite effect on BxPC-3 cells (Figure 5C, 5D). Collectively, the expression of MRGBP contributes to the migration and invasion of PDAC.

### Overexpression of MRGBP induces EMT in PDAC cells

**Figure 6:**
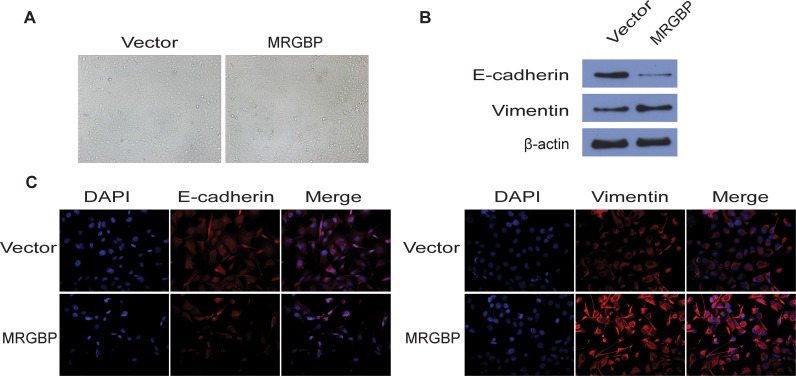
Overexpression of MRGBP induces EMT in PDAC cells **(A)** The pictographs show morphology of BxPC-3 cells with vector alone control and with MRGBP overexpression. **(B)** Western blotting analysis for expression of EMT related markers in BxPC-3 cells with vector alone control and MRGBP overexpression. **(C)** Immunofluorescence was used to detect the fluorescence intensity of E-cadherin and Vimentin in BxPC-3vector and BxPC-3MRGBP cells.

The epithelial-to-mesenchymal transition (EMT), which is characterized by the down-regulation of E-cadherin and overexpression of Vimentin, is a critical biological process in tumor invasion, metastasis and chemo-resistance. To investigate the effect of MRGBP on EMT, BxPC-3 cells overexpressing human MRGBP were generated via lentivirus transduction. Phenotypically, compared to the empty vector, MRGBP overexpression led to morphological changes in BxPC-3 cells such as spindle-like cell shape and loss of cell-to-cell adhesion (Figure 6A). In addition, EMT markers were detected by Western blot and Immunofluorescence staining. From the results of the above two experiments, we observed that MRGBP overexpression in BxPC-3 cells induced an increased expression of mesenchymal marker Vimentin, whereas a decreased expression of epithelial marker E-cadherin (Figure 6B, 6C). Taken together, these data revealed that upregulated expression of MRGBP induces EMT in PDAC cells.

## DISCUSSION

Pancreatic ductal adenocarcinoma (PDAC) is a highly lethal disease which is difficult to diagnose at early stage. Although many potential diagnostic and therapeutic biomarkers have been identified in pancreatic cancer, additional novel molecular targets are still urgently required.

In this study, we observed that MRGBP expression was commonly upregulated in PDAC tissues comparing with adjacent noncancerous pancreatic tissues and normal pancreas, and was strongly associated with survival in pancreatic cancer from two independent GEO datasets. The current study provides insights into the oncogenic role of MRGBP in PDAC through both *in vitro* and *in vivo* experiments. Our results strongly suggested that MRGBP promote PDAC progression, which is similar to the previous studies in other types of cancer, such as prostate cancer, colorectal cancer, cervical cancer and squamous cell carcinoma [9–12]. Our observation also showed that knockdown of MRGBP can reduce tumor growth and induced apoptosis in PDAC cells *in vitro*, which may play an important role in proliferation of cancer cells. Meanwhile, elevated expression of MRGBP was an indicator for the poor prognosis and induces EMT in PDAC cells.

So far, little has been known about the expression pattern and cellular functions of MRGBP in pancreatic cancer. Previous reports have shown that expression of MRGBP was upregulated in colorectal carcinoma and might be associated with the transformation from adenoma to carcinoma in colorectal carcinogenesis [9, 14]. Scholars suggested that MRGBP has an important function in proliferation of cancer cells through the regulation of bromodomain containing 8 (BRD8) [9]. In their study, 41 genes were downregulated by MRGBP siRNA, some of which were responsible for DNA replication, such as CDT1, MCM2, MCM5, and MCM7 [14]. CDT1 and MCM proteins have been shown to be essential for DNA replication. It has been reported that CDT1 function as an oncogene in several types of human cancers [15–17]. Elizabeth et al supported overexpression of CDT1 could override the negative control of geminin, which is a negative regulator of DNA replication by targeting CDT1 [14]. Meanwhile, Tatsumi et al have demonstrated that the overexpression of CDT1 induced re-replication and/or chromosomal damage, leading to chromosomal instability, which might be a new mechanism of carcinogenesis [17, 18]. Additionally, MCM family functions as a modulator on promoting the process of DNA replication and is also identified to be potential biomarkers of numerous malignancies, including pancreatic cancer [19, 20]. Furthermore, it has been shown that some members of MCMs were abnormally upregulated in diverse cancers, and higher MCMs expression could enhance the proliferation of malignant cells and predict poor prognosis [21, 22]. So it is logical to draw that MRGBP dysregulation may take part in induction of carcinogenesis via regulating the expression of CDT1 and MCMs.

However, there may be other possible mechanisms to illustrate the same problem. Expression of MRGBP has been shown to play an important role in regulating stability and/or synthesis of MRG15 and MRGX protein, which are two stable components of both TIP60/HAT complex and HDACs complexes [5, 13]. It has been reported that TIP60/HAT complex not only controls chromatin structure and regulates transcription through its HAT activity, but also functions as a signaling platform, including replication, DNA repair, cell cycle progression, stem cell maintenance and differentiation, and cell migration and invasion [23–25]. Importantly, TIP60 has been implicated as a tumor suppressor in diverse cancers by controlling p53-dependent transcriptional activity [6, 26–28]. Conversely, the overexpression of HDACs were already been confirmed in a wide variety of tumor tissues, which correlate with tumor occurrence and advance, as well as aggressive biological behaviors [7, 8, 29–32]. Stojanovic et al. demonstrated that HDAC1 and HDAC2 contribute to maintain the expression of p53 mutants and weaken its tumour-suppressive functions [7]. In addition, the expression of many oncogenes are regulated by HDACs and significantly reduced by HDAC inhibitor (HDACi), such as oncogenic JNK, KRAS, CIP2A, MKK7, Raf1, ERBB2, MITF, MYC, MYCN and EGFR [29–37]. These possible mechanisms seem to be contradictory and need to be further investigated.

In conclusion, this study demonstrates for the first time that accumulation of MRGBP contributes to proliferation and metastasis of PDAC. Further wide-ranging exploration of the precise role of MRGBP in promoting the proliferation and metastasis will be needed to uncover in detail. Furthermore, MRGBP is a potential diagnostic and prognostic biomarker for PDAC, and may be a novel therapeutic target for PDAC patients.

## MATERIALS AND METHODS

### Clinical materials

The matched clinical tissue samples of primary PDAC and adjacent normal pancreatic ductal epithelial from the same patient were collected from 58 patients (33 were male and 25 were female) of PDAC undergoing surgical resection at the Zhongnan Hospital of Wuhan University, Renmin hospital of Wuhan University or Cancer Hospital of Hubei Province from January 2010 to November 2013. Adjacent normal pancreatic ductal epithelial specimens were obtained from sites 5–10cm apart from the primary tumors. None of the patients had received chemotherapy or radiotherapy before tumor resection. Informed consent was obtained from all patients, and the study was approved by the Ethics Committee of Zhongnan Hospital of Wuhan University.

### Cell culture and reagents

Human PDAC cell lines (PANC-1, SW1990, Mia PaCa-2, AsPC-1, BxPC-3, CFPAC1) and human normal pancreatic ductal epithelial (HPNE) cells were purchased from the Type Culture Collection of the Chinese Academy of Sciences (Shanghai, China). All of the cell lines were routinely maintained in Dulbecco's modified Eagle medium (DMEM; Gibco, Carlsbad, CA) containing 10% fetal bovine serum (FBS), 100 mg/ml penicillin, and 100 mg/ml streptomycin at 37°C/5% CO_2_. Antibodies to MRGBP, E-cadherin, Vimentin, and β-actin were purchased from Santa Cruz Biotechnology (Dallas, TX). Anti-rabbit horseradish peroxidase (HRP)-labeled secondary antibody was obtained from Promega Biotechnology (Dallas, TX). Cell Counting Kit-8 (CCK-8) was purchased from DoJinDo (Japan).

### Immunohistochemical staining

The expression of MRGBP in pancreatic tumors was evaluated by immunohistochemical staining (IHC) using anti-MRGBP polyclonal antibody. The 58 cases of human PDAC tissues in the evaluation cohort were used for IHC. Formalin-fixed, paraffin-embedded tissue sections (5 μm) were first stained with hematoxylin and eosin (HE) for histological examination. Subsequently, antigen retrieval was performed by microwaving and citrate buffer (pH 6.0). Endogenous peroxidase activity was blocked by incubation with 3% H_2_O_2_ for 15 minutes at room temperature. After washing with phosphate-buffered saline (PBS), the sections were overnight incubated with primary antibody at 4°C. Then, sections were incubated for 20 minutes at room temperature with HRP-conjugated anti-rabbit IgG. Finally, the sections were lightly counterstained with hematoxylin.

### Western blot analysis

Whole cell lysates were prepared in buffer containing 150 mM NaCl, 50 mM Tris (pH 8.0), 1% NP-40, 0.5% sodium deoxycholate, 50 mM NaF, 0.1% SDS, 1 mM Na_3_VO_4_. The protein concentration of the supernatant was measured by the BCA protein assay (Pierce Biotechnology). Proteins were resolved by 10% SDS-PAGE and transferred onto nitrocellulose membrane (Millipore, Billerica, MA). The membranes were incubated with primary antibodies and species- specific HRP-labeled secondary antibody. The bands were detected using the chemiluminescence kit phototope-HRP kit (Pierce).

### RNA isolation and real-time quantitative PCR analysis

Total RNA was isolated from cell lines and tissues using TRIzol (Invitrogen), following the manufacturer's instructions. cDNA was synthesized using a high capacity cDNA reverse transcription kit (Applied Biosystems). For the quantification of gene amplification, Real-time quantitative PCR (qRT-PCR) was performed using SYBR Green PCR Master Mix (TaKaRa, Ohtsu, Japan) on an Applied Biosystems 7900HT real-time PCR system. MRGBP primers (5′-ATTCTTCCATTCCCGAATCC-3′ and 5′-CCCAAACTCCCTGAAGATGA-3′) [9] were used for qRT-PCR. GAPDH was used as for MRGBP normalization.

### Transient transfection and lentiviral constructs

Transfection of vectors, or small interfering RNA (siRNA) into PDAC cells was performed with Lipofectamine 2000 reagent (Invitrogen, Carlsbad, CA) following the manufacturer's instructions. Transfected cells were incubated for 48h, followed by cell harvesting and analysis. The siRNAs against the human MRGBP gene were designed and purchased commercially from Genepharma Co., Ltd, Shanghai, China. The sequences targeting MRGBP are as follows; si-MRGBP-1 forward: 5′-CCAGCAAAGACAAAGAGAATT-3′; si-MRGBP-1 reverse: 5′-UUCUCUUUGUCUUUGCUGGTT-3′; si-MRGBP-2 forward: 5′-GCAAAGACAAAGAG AAGAATT-3′; si-MRGBP-2 reverse: 5′-UUCU UCUCUUUGUCUUUGCTT-3′; control siRNA forward: 5′-UUCUCCGAACGUGUCACGUTT-3′; control siRNA reverse: 5′-ACGUGACACGUUCGGAGAATT-3′; qRT-PCR or Western blot analysis was employed to evaluate transfection efficiency. For establishment of stable MRGBP-overexpressing cells, lentiviral vector encoding human MRGBP cDNA was constructed by Inovogen, Beijing, China and designated as pLV-MRGBP. The empty vector was used as negative control, designated as pLV-vector.

### Cell proliferation assay

Cells from each group were seeded in 96-well plates containing 100 ul medium and 10% FBS. 10 ul Cell Counting Kit-8 (CCK-8) (Dojindo, Tokyo, Japan) reagent was added at 24 h after seeding and cultured at 37°C and 5% for 1 h. The data of optical density (OD) value was measured by a multifunctional microplate reader (Bio-Rad) at 450nm. The growth curve was drawn with time as abscissa, and the absorbance value as ordinate.

### Plate colony formation assay

Plate colony formation assay was performed to evaluate anchorage-independent growth. Cells (1×10^3^) at a log phase of growth were trypsinized and resuspended in 1.0ml of DMEM medium containing 10% FBS and 0.33% agar (Sigma) and plated in the bottom of 6-well plates containing the same medium with 0.5% agar. Then the cells were allowed to attach for 14days at 37°C and 5% CO_2_ incubator. Cells were washed 3 times with PBS, fixed with 4% paraformaldehyde at room temperature for 25 minutes, rinsed again with PBS, and then stained with 0.1% crystal violet for 30 minutes at room temperature. Finally, colonies were counted under a microscope.

### Cell migration and invasion assays

The cell migration and invasion assays were measured by transwell model (Corning, NY) according to the manufacturer's instructions. In total, serum-free DMEM media containing tumor cells was seeded into the upper chamber, while medium supplemented with 10% FBS was placed in the lower chamber as a chemo-attractant. Following incubation at 37°C for 48 h, the cells on the top side of the inserts were removed gently with a cotton swab. Subsequently, the cells that were located on the lower surface of membrane were fixed by 4% paraformaldehyde, stained with 0.1% crystal violet, and counted under an inverted microscope. For the transwell migration assay, the remaining protocol was similar to the cell migration assay, except that the transwell membranes were pre-coated with matrigel.

### Cell apoptosis analysis

Annexin V-FITC/PI staining was employed to discover whether MRGBP regulates the apoptosis of PDAC cells. Cells were harvested in complete DMEM medium and were collected by centrifuging at 1000rpm for 5 min. Subsequently, cell apoptosis was assayed by staining with propidium iodide (PI) and Annexin V-FITC (BD Pharmingen, USA) following the manufacturer's instructions. The apoptosis of PDAC cells was detected by flow cytometer.

### Animal experiments

Animal studies were conducted according to the Chinese national guidelines for the care and use of laboratory animals. 6-week-old male BALB/C nude mice were employed and housed in pathogen-free conditions. A total of 3×10^6^ Mia PaCa-2 cells (MRGBP-control, si-MRGBP-1 and si-MRGBP-2) were suspended in 100ul PBS and injected subcutaneously in the right scapular region of mice. On day 21, animals were sacrificed and tumors were excised and weighted. Tumor volume was calculated by the following formula: V = 1/2 (width × length × height).

### Immunofluorescent staining

For immunofluorescent staining, BxPC-3 cells were plated at glass coverslips. After washing with PBS extensively, the cell-bearing coverslips were fixed in 4% paraformaldehyde for 15 min at room temperature, and then rinsed with PBS for 3 times. Cells were permeabilized with 0.1% Triton X-100 in PBS for 10 min, washed 3 times with PBS, and blocked with 5% bovine serum albumin for 1 h. Then the coverslips were incubated with diluted primary antibody against target protein (E-cadherin or Vimentin) at 4°C overnight. After washed 3 with PBS, the coverslips finally co-incubated with fluorescence-labeled goat anti-mouse IgG at room temperature for 1h in darkness. The nuclei was stained with 4′, 6-diamidino-2-phenylindole (DAPI). Fluorescent images were captured by using a fluorescence microscope (Olympus, Tokyo, Japan).

### Statistical analysis

Overall survival (OS) was defined as the time from the date of the diagnosis to the date of death from any cause or the last contact, i.e., the date of the last follow-up. The patients who were alive at the last follow-up evaluation were censored. Survival curves were evaluated using the Kaplan-Meier method and the statistical differences between survival curves were tested by the log-rank test. Chi-square test or Student's t-test was performed to evaluate the correlation between gene expression and the clinicopathologic features. All means were calculated from at least three independent experiments in cellular studies, and results were carried out to determine the statistical significance using the two-tailed, unparied Student's t-test. The error bars represented standard error. Statistical significance was set at P < 0.05. SPSS software (version 13.0; SPSS, Inc., Chicago, IL, USA) was used for statistical analysis. Graphical representations were performed with GraphPad Prism 5 software (San Diego, CA).

## References

[1] Torre LA, Bray F, Siegel RL, Ferlay J, Lortet-Tieulent J, Jemal A (2015). Global cancer statistics, 2012. CA Cancer J Clin.

[2] Rahib L, Smith BD, Aizenberg R, Rosenzweig AB, Fleshman JM, Matrisian LM (2014). Projecting cancer incidence and deaths to 2030: the unexpected burden of thyroid, liver, and pancreas cancers in the United States. Cancer Res.

[3] Siegel RL, Miller KD, Jemal A (2015). Cancer statistics, 2015. CA Cancer J Clin.

[4] Garrido-Laguna I, Hidalgo M (2015). Pancreatic cancer: from state-of-the-art treatments to promising novel therapies. Nat Rev Clin Oncol.

[5] Cai Y, Jin J, Tomomori-Sato C, Sato S, Sorokina I, Parmely TJ, Conaway RC, Conaway JW (2003). Identification of new subunits of the multiprotein mammalian TRRAP/TIP60-containing histone acetyltransferase complex. J Biol Chem.

[6] Sakuraba K, Yokomizo K, Shirahata A, Goto T, Saito M, Ishibashi K, Kigawa G, Nemoto H, Hibi K (2011). TIP60 as a potential marker for the malignancy of gastric cancer. Anticancer Res.

[7] Stojanovic N, Hassan Z, Wirth M, Wenzel P, Beyer M, Schäfer C, Brand P, Kroemer A, Stauber RH, Schmid RM, Arlt A, Sellmer A, Mahboobi S (2016). HDAC1 and HDAC2 integrate the expression of p53 mutants in pancreatic cancer. Oncogene.

[8] Edderkaoui M, Xu S, Chheda C, Morvaridi S, Hu RW, Grippo PJ, Mascainas E, Principe DR, Knudsen B, Xue J, Habtezion A, Uyeminami D, Pinkerton KE, Pandol SJ (2016). HDAC3 mediates smokion-induced pancreatic cancer. Oncotarget.

[9] Yamaguchi K, Sakai M, Shimokawa T, Yamada Y, Nakamura Y, Furukawa Y (2010). C20orf20 (MRG-binding protein) as a potential therapeutic target for colorectal cancer. Br J Cancer.

[10] Scotto L, Narayan G, Nandula SV, Arias-Pulido H, Subramaniyam S, Schneider A, Kaufmann AM, Wright JD, Pothuri B, Mansukhani M, Murty VV (2008). Identification of copy number gain and overexpressed genes on chromosome arm 20q by an integrative genomic approach in cervical cancer: potential role in progression. Genes Chromosomes Cancer.

[11] Ito S, Ueda T, Ueno A, Nakagawa H, Taniguchi H, Kayukawa N, Miki T (2014). A genetic screen in Drosophila for regulators of human prostate cancer progression. Biochem Biophys Res Commun.

[12] Watt SA, Pourreyron C, Purdie K, Hogan C, Cole CL, Foster N, Pratt N, Bourdon JC, Appleyard V, Murray K, Thompson AM, Mao X, Mein C (2011). Integrative mRNA profiling comparing cultured primary cells with clinical samples reveals PLK1 and C20orf20 as therapeutic targets in cutaneous squamous cell carcinoma. Oncogene.

[13] Hayakawa T, Ohtani Y, Hayakawa N, Shinmyozu K, Saito M, Ishikawa F, Nakayama J (2007). RBP2 is an MRG15 complex component and downregulates intragenic histone H3 lysine 4 methylation. Genes Cells.

[14] Yamaguchi K, Sakai M, Kim J, Tsunesumi S, Fujii T, Ikenoue T, Yamada Y, Akiyama Y, Muto Y, Yamaguchi R, Miyano S, Nakamura Y, Furukawa Y (2011). MRG-binding protein contributes to colorectal cancer development. Cancer Sci.

[15] Liontos M, Koutsami M, Sideridou M, Evangelou K, Kletsas D, Levy B, Kotsinas A, Nahum O, Zoumpourlis V, Kouloukoussa M, Lygerou Z, Taraviras S, Kittas C (2007). Deregulated overexpression of hCdt1 and hCdc6 promotes malignant behavior. Cancer Res.

[16] Li JN, Feng CJ, Lu YJ, Li HJ, Tu Z, Liao GQ, Liang C (2008). mRNA expression of the DNA replication-initiation proteins in epithelial dysplasia and squamous cell carcinoma of the tongue. BMC Cancer.

[17] Tatsumi Y, Sugimoto N, Yugawa T, Narisawa-Saito M, Kiyono T, Fujita M (2006). Deregulation of Cdt1 induces chromosomal damage without rereplication and leads to chromosomal instability. J Cell Sci.

[18] Vaziri C, Saxena S, Jeon Y, Lee C, Murata K, Machida Y, Wagle N, Hwang DS, Dutta A (2003). A p53-dependent checkpoint pathway prevents rereplication. Mol Cell.

[19] Maiorano D, Lutzmann M, Mechali M (2006). MCM proteins and DNA replication. Curr Opin Cell Biol.

[20] Peng YP, Zhu Y, Yin LD, Zhang JJ, Guo S, Fu Y, Miao Y, Wei JS (2016). The expression and prognostic roles of MCMs in pancreatic cancer. PLoS One.

[21] Hua C, Zhao G, Li Y, Bie L Minichromosome Maintenance (MCM) Family as potential diagnostic and prognostic tumor markers for human gliomas. BMC Cancer.

[22] Kwok HF, Zhang SD, McCrudden CM, Yuen HF, Ting KP, Wen Q, Khoo US, Chan KY (2015). Prognostic significance of minichromosome maintenance proteins in breast cancer. Am J Cancer Res.

[23] Murr R, Loizou JI, Yang YG, Cuenin C, Li H, Wang ZQ, Herceg Z (2006). Histone acetylation by Trrap-Tip60 modulates loading of repair proteins and repair of DNA double-strand breaks. Nat Cell Biol.

[24] Wurdak H, Zhu S, Romero A, Lorger M, Watson J, Chiang CY, Zhang J, Natu VS, Lairson LL, Walker JR, Trussell CM, Harsh GR, Vogel H (2010). An RNAi screen identifies TRRAP as a regulator of brain tumor-initiating cell differentiation. Cell Stem Cell.

[25] Loukopoulos P, Shibata T, Katoh H, Kokubu A, Sakamoto M, Yamazaki K, Kosuge T, Kanai Y, Hosoda F, Imoto I, Ohki M, Inazawa J, Hirohashi S (2007). Genomewide array-based comparative genomic hybridization analysis of pancreatic adenocarcinoma: identification of genetic indicators that predict patient outcome. Cancer Sci.

[26] Bassi C, Li YT, Khu K, Mateo F, Baniasadi PS, Elia A, Mason J, Stambolic V, Pujana MA, Mak TW, Gorrini C (2016). The acetyltransferase Tip60 contributes to mammary tumorigenesis by modulating DNA repair. Cell Death Differ.

[27] Pandey AK, Zhang Y, Zhang S, Li Y, Tucker-Kellogg G, Yang H, Jha S (2015). TIP60-miR-22 axis as a prognostic marker of breast cancer progression. Oncotarget.

[28] Sho T, Tsukiyama T, Sato T, Kondo T, Cheng J, Saku T, Asaka M, Hatakeyama S (2011). TRIM29 negatively regulates p53 via inhibition of Tip60. Biochim Biophys Acta.

[29] Marks P, Rifkind RA, Richon VM, Breslow R, Miller T, Kelly WK (2001). Histone deacetylases and cancer: causes and therapies. Nat Rev Cancer.

[30] Bousquet MS, Ma JJ, Ratnayake R, Havre PA, Yao J, Dang NH, Paul VJ, Carney TJ, Dang LH, Luesch H (2016). Multidimensional screening platform for simultaneously targeting oncogenic KRAS and hypoxia-inducible factors pathways in colorectal cancer. ACS Chem Biol.

[31] Balliu M, Cellai C, Lulli M, Laurenzana A, Torre E, Vannucchi AM, Paoletti F (2016). HDAC1 controls CIP2A transcription in human colorectal cancer cells. Oncotarget.

[32] Giaginis C, Damaskos C, Koutsounas I, Zizi-Serbetzoglou A, Tsoukalas N, Patsouris E, Kouraklis G, Theocharis S (2015). Histone deacetylase (HDAC)-1,-2,-4 and -6 expression in human pancreatic adenocarcinoma: associations with clinicopathological parameters, tumor proliferative capacity and patients’ survival. BMC Gastroenterol.

[33] Chou CW, Wu MS, Huang WC, Chen CC (2011). HDAC inhibition decreases the expression of EGFR in colorectal cancer cells. PloS One.

[34] LaBonte MJ, Wilson PM, Fazzone W, Russell J, Louie SG, El-Khoueiry A, Lenz HJ, Ladner RD (2011). The dual EGFR/HER2 inhibitor lapatinib synergistically enhances the antitumor activity of the histone deacetylase inhibitor panobinostat in colorectal cancer models. Cancer Res.

[35] Scott GK, Marx C, Berger CE, Saunders LR, Verdin E, Schafer S, Jung M, Benz CC (2008). Destabilization of ERBB2 transcripts by targeting 3′ untranslated region messenger RNA associated HuR and histone deacetylase-6. Mol Cancer Res.

[36] Yokoyama S, Feige E, Poling LL, Levy C, Widlund HR, Khaled M, Kung AL, Fisher DE (2008). Pharmacologic suppression of MITF expression via HDAC inhibitors in the melanocyte lineage. Pigment Cell Melanoma Res.

[37] Sharma V, Koul N, Joseph C, Dixit D, Ghosh S, Sen E (2010). HDAC inhibitor, scriptaid, induces glioma cell apoptosis through JNK activation and inhibits telomerase activity. J Cell Mol Med.

